# Associations between endothelial dysfunction and clinical and laboratory parameters in children and adolescents with sickle cell anemia

**DOI:** 10.1371/journal.pone.0184076

**Published:** 2017-09-01

**Authors:** Rozana Santos Teixeira, Regina Terse-Ramos, Tatiane Anunciação Ferreira, Vinícius Ramos Machado, Marya Izadora Perdiz, Isa Menezes Lyra, Valma Lopes Nascimento, Ney Boa-Sorte, Bruno B. Andrade, Ana Marice Ladeia

**Affiliations:** 1 Bahiana School of Medicine and Public Health, Bahia Foundation for the Development of Sciences Salvador, Salvador, Bahia, Brazil; 2 School of Medicine, Federal University of Bahia, Salvador, Bahia, Brazil; 3 University Salvador (UNIFACS), Laureate International Universities, Salvador, Bahia, Brazil; 4 Hematology and Hemotherapy Foundation of Bahia, Salvador, Bahia, Brazil; 5 Instituto Gonçalo Moniz, Fundação Oswaldo Cruz, Salvador, Bahia, Brazil University of Salvador, Salvador, Bahia, Brazil; 6 Multinational Organization Network Sponsoring Translational and Epidemiological Research (MONSTER) Initiative, Fundação José Silveira, Salvador, Bahia, Brazil; 7 Catholic University of Salvador, Salvador, Bahia, Brazil; Université Claude Bernard Lyon 1, FRANCE

## Abstract

**Background:**

Hematological changes can drive damage of endothelial cells, which potentially lead to an early endothelial dysfunction in patients with sickle cell anemia (SCA). An association may exist between endothelial dysfunction and several clinical manifestations of SCA. The present study aims to evaluate the links between changes in endothelial function and clinical and laboratory parameters in children and adolescents with SCA.

**Methods:**

This study included 40 children and adolescents with stable SCA as well as 25 healthy children; aged 6–18 years. All study subjects were evaluated for endothelial function using Doppler ultrasonography. In addition, a number of laboratory assays were performed, including reticulocyte and leukocyte counts as well as measurement of circulating levels of total bilirubin, C-reactive protein (CRP), glucose, lipoproteins and peripheral oxyhemoglobin saturation. These parameters were also compared between SCA patients who were undertaking hydroxyurea (HU) and those who were not.

**Results:**

Flow-mediated vasodilation (FMD) values were found to be reduced in SCA patients compared with those detected in healthy controls. SCA individuals with lower FMD values exhibited higher number of hospital admissions due to vaso-occlusive events. Additional analyses revealed that patients who had decreased FMD values exhibited higher odds of acute chest syndrome (ACS) episodes. A preliminary analysis with limited number of individuals failed to demonstrate significant differences in FMD values between SCA individuals who were treated with HU and those who were not.

**Conclusions:**

Children and adolescents with SCA exhibit impaired endothelial function. Reductions in FMD values are associated with ACS. These findings underline the potential use of FMD as screening strategy of SCA patients with severe prognosis at early stages.

## Introduction

Sickle cell anemia (SCA) belongs to a group of hereditary hemoglobinopathies and has great importance due to its wide geographic distribution, diverse clinical manifestations, and impact on mortality, quality of life and public health spending [1]. Extensive research on SCA vasculopathy has demonstrated that acute and chronic manifestations observed in this disease result from complex multifactorial mechanisms involving endothelial dysfunction. In SCA patients, abnormal red blood cells activate endothelial cells, leukocytes and platelets, driving expression of adhesion molecules such as VCAM-1, ICAM-1 and E-selectin as well as of pro-inflammatory cytokines including TNF-α and IL-6 [2,3]. The degree of endothelial activation observed in SCA patients appears to be a contributing factor for the formation of cellular clusters which interact with the vessel wall and contribute to vascular occlusion [2,4–6].

Nitric oxide (NO) is a critical factor responsible for maintaining the vascular tone [7], by antagonizing potent vasoconstrictors derived from endothelial cells, such as angiotensin II and endothelin-1, while also inhibiting the activation of platelets and leukocytes [8]. In SCA, NO is described to be less bioavailable for at least three important reasons. First, the destruction of red blood cells leads to release of free hemoglobin [9,10], which scavengers NO. Second, sustained hemolysis observed in SCA triggers expression of arginase-1, which in turn is associated with decreased NO production and substantial increase in production of ornithine, polyamines and prolines [5,11]. This latter phenomenon is described to induce smooth muscle proliferation, collagen deposition and vascular stenosis [9,10]. The third mechanism is the excessive production of reactive oxygen species (ROS), observed during hemolysis, which promotes oxidative stress and perturbs the NO homeostasis [12].

Both the pro-inflammatory environment and the reduction of NO bioavailability potentially drive endothelial dysfunction in SCA. Such condition is characterized by an impaired endothelium-dependent vasodilation response, which promotes platelet aggregation, adhesion of leukocytes and proliferation of smooth muscle cells [13]. A previous study has shown that endothelial function reflects vascular health [14]. In the present study, we tested the hypothesis that children and adolescents with SCA present changes in their endothelial function when compared to healthy children and adolescents without hemoglobinopathies. In addition, we evaluated the association between endothelial dysfunction status and clinical and laboratory parameters.

## Methods

### Ethics statement

All clinical investigations were conducted according to the principles expressed in the Declaration of Helsinki. Written informed consent was obtained from each participant or legal guardian at the study enrollment. This study was approved by the Ethics Committee of the Bahiana School of Medicine and Public Health, (protocol number: 568.913/2014). Access to the registry data was authorized by the boards of the participating institutions.

### Study design

The main study population was composed of children and adolescents diagnosed with SS hemoglobinopathy. The admission criteria for patients with SCA were: age 6–18 years, presence of hemoglobin (Hb) SS diagnosed by hemoglobin electrophoresis and/or high-performance liquid chromatography, and absence of acute complications or infectious conditions in a period of at least one month before inclusion. Overall exclusion criteria were acute infectious identified at the study screening, dyslipidemia, or obesity. In addition, we excluded individuals presenting with circulating levels of C-reactive protein (CRP) above 10mg/L, given the reported strong association with systemic inflammation linked to cardiovascular disease [15].

A second study group included healthy individuals with the following inclusion criteria: similar age range, absence of the diagnosis of acute or chronic diseases, presence of Hb AA determined by Hb electrophoresis and/or high-performance liquid chromatography, absence of infections in at least one month prior to inclusion, and body mass index/age ratio values below +2 Z-score in the growth curves from the World Health Organization (WHO).

SCA subjects were enrolled and followed-up at 2 referral centers for hematological diseases: (i) the Magalhães Neto Hematology Outpatient Clinic, Federal University of Bahia, Brazil and (ii) the Hematology and Hemotherapy Foundation of Bahia, Brazil. Children included in the healthy control group were enrolled at the general pediatric teaching and healthcare outpatient clinic of the Roberto Santos General Hospital, Bahiana School of Medicine and Public Health, as well as at the outpatient clinic for adolescents at the Magalhães Neto Hematology Outpatient Clinic, Federal University of Bahia, Brazil.

Clinical evaluation of SCA patients included a questionnaire with information regarding number of vaso-occlusive crisis (VOC), number of episodes in which the participants needed emergency care due to VOC, number of hospitalizations due to VOC, number of episodes of acute chest syndrome (ACS) evaluated over the last year; length of hospital stay (days) due to ACS; occurrence of pneumonia, osteomyelitis, priapism, osteonecrosis, splenic sequestration, and splenectomy; number of prior transfusions until the time of the study, and HU use as therapeutic approach.

The physical examination included measurement of weight and height to calculate body mass index (kg/m^2^), which was also expressed according to the WHO Z-score system [16]; oxyhemoglobin peripheral saturation (SpO_2_) measurement; and systolic and diastolic blood pressure determination. In laboratory tests, the following were assessed: hematocrit (%), hemoglobin (g/dL), mean corpuscular volume (fL), mean corpuscular hemoglobin concentration (pg), leukocyte count (×10^3^/L), platelets (×10^3^/L), reticulocytes (%), lactate dehydrogenase (U/L), transaminases (U/L), total bilirubin (mg/dL), indirect and direct bilirubin (mg/dL), total cholesterol (mg/dL), low-density lipoprotein-cholesterol (LDL-C; mg/dL), high-density lipoprotein-cholesterol (HDL-C; mg/dL), triglycerides (mg/dL), glucose (mg/dL), and high-sensitivity CRP (mg/L).

### Sample size calculation and study power

Sample size was calculated based on a standard deviation (SD) of the brachial artery dilation average of 0.2 to detect a 0.25 difference, as obtained in a previous study [17]. Considering an alpha error of 0.05 and a study power of 90%, the we found that the study needed a total of 30 subjects, 15 with HbSS and 15 without hemoglobinopathies. Considering the technical difficulties associated with the pediatric age group in question, in order to reduce the risk of loss, we categorized 60 subjects into 3 groups: 20 in HU use, 20 in HU non-use, and 20 in without hemoglobinopathies. The calculation was performed using the WinPepi^®^ statistical software.

### Laboratory assays

Blood samples were collected by a qualified technician for laboratory analyses. For each study participant, 10 mL of blood were collected by venipuncture, after a minimum 12-h fasting, in untreated tubes for biochemical analysis and in ethylenediaminetetracetic acid (EDTA) tubes for complete blood count. The levels of total cholesterol (TC), HDL cholesterol, LDL cholesterol and triglycerides (TG) were measured using enzymatic methods in a reference laboratory. High-sensitivity quantitative CRP was measured using turbidimetry.

### Event definitions

The various clinical manifestations were defined as follows. VOC [18] was described as pain symptoms warranting analgesia while VOC with physical disability was defined as VOC that restricted work or attending school. ACS [19–21] was defined as new pulmonary infiltrate involving at least one segment of the lung, isolated atelectasis with one or more associated respiratory symptoms, and/or hypoxemia (PaO_2_ <60 mmHg or SpO_2_ below 2% of basal). Priapism was defined as unwanted and persistent painful erection lasting more than two to four hours. Splenic sequestration [22] was defined as hemoglobin reduction by at least 20% of baseline Hb, associated with an enlarged palpable spleen size of at least 2 cm from the baseline.

### Assessment of endothelial function

To evaluate endothelial function, a protocol established under the guidelines for ultrasonographic evaluation of the forearm was used [23]. The examinations were performed at the Cardiovascular Research Laboratory, Bahiana School of Medicine and Public Health, Salvador, Brazil, using the VIVID 3 ultrasound scanner (GE Healthcare) 12 MHz multi-frequency transducer. The endothelium-dependent vasomotor function was assessed using flow-mediated vasodilation (FMD) and measured using reactive hyperemia. The examinations were performed on patients who fasted for at least 8 h after 30 min of rest, with the room temperature set to 20–25°C. To avoid circadian variations, all examinations were performed in the morning. The patients were examined in the supine position, with the right arm positioned ergonomically. The mercury sphygmomanometer cuff, which acts as a pneumatic tourniquet, was placed on the right forearm, below the elbow. The transducer was placed on anterior region of right arm above the antecubital fossa. The brachial artery was identified in longitudinal section and later, the center and lumen-intima interface of the anterior and posterior wall of the vessel were also identified; The Doppler sample was placed at an angle of 60°, adjusting the Doppler's grayscale, depth, filter, and scale settings. The increased flow was induced by inflation of the cuff around the arm up to 250 mmHg for 4 min with continuous monitoring of the arterial image. The cuff was then deflated leading to reactive hyperemia, with continuous monitoring of the first 5 flows and diameter of the artery for 120 s (diameter measurement after 60 s).Once the best image for analysis was chosen and the borders of the artery walls were identified, three measurements of the arterial diameter were performed. The mean of these measurements represented the final diameter of the brachial artery at each stage of the evaluation.The examinations were performed by a specifically trained physician with proven experience in the technique [24–26]. Throughout the examination, a synchronized electrocardiogram was obtained.

### Statistical analysis

For descriptive analysis, quantitative variables were represented as mean and standard deviations (SD) when they exhibited Gaussian distribution, and as medians and interquartile ranges (IQR) when they exhibited non-Gaussian distributions. To evaluate the Gaussian distribution of variables, the Kolmogorov-Smirnov and Shapiro-Wilk normality tests were applied. Categorical variables were represented as simple frequencies and percentages. To compare categorical variables using bivariate analysis, the chi-square test or Fisher's exact test was used. To compare the means, we used the Student’s *t* test for independent samples or Mann-Whitney *U* test to compare medians of variables with non-parametric distribution. Correlations were tested using the Spearman rank test. To compare the means of numeric variables with more than 2 categorized groups, we used ANOVA with Bonferroni or Kruskal-Wallis correction for ranking comparison, if the distribution was non-parametric. Receiver Operator Characteristics (ROC) curves were employed to test the accuracy of potentially using FMD values to distinguish SCA patients from healthy as well as SCA individuals presenting with ACS history from those without.

Multivariate analysis using a linear regression model, adjusted for age and gender was performed to investigate factors associated ACS history. The analyses were conducted using the, Graphpad Prism 7.0 (GraphPad Software, San Diego, CA) and IBM Statistical Package for the Social Sciences (SPSS ^®^, Chicago, IL, EUA. 20.0).

## Results

The study population consisted of 40 patients with SCA (HbSS) and 25 healthy children and adolescents aged 12.3 ± 3 years (age range, 6–17 years) and 11.4 ± 3 years (age range, 6–18 years), respectively. In the SCA group, 18 patients were taking HU at study enrollment. SCA patients were more frequently male than healthy individuals (n = 24, [60%] vs. n = 7 [28%], respectively, p < 0.001). The groups differed with regard to values of hemoglobin, leukocyte counts, platelet counts, reticulocyte counts, serum levels of lactate dehydrogenase, glutamic-oxaloacetic transaminase, total bilirubin, high-sensitivity quantitative CRP, as well as oxygen saturation (Table 1). In addition, SCA patients had lower circulating levels of total cholesterol, LDL cholesterol, and HDL cholesterol and higher concentrations of triglycerides than healthy persons (Table 1). Furthermore, BMI values were on average lower in the group of patients with SCA compared to that in the control group (16.4 ± 2 kg/m^2^ in SCA vs. 18.5 ± 3.8 kg/m^2^ in healthy controls, p = 0.02). Similar results were obtained with z-scored BMI values (SCA: -1 ± 0.87 vs. healthy: and -0.07 ± 1.29, p = 0.02) (Table 1).

**Table 1:** Sociodemographic and clinical characteristics study groups.

Characteristic	Sickle cell anemia	healthy control	*P-value*
N	40	25	
Age (years)	12.3 ± 3	11.4 ± 3	0.24
Male n (%)^a^	24 (60)	7 (28)	0.001
SpO_2_ (%)	95 ± 2.9	98 ± 0.7	<0.001
SBP (mmHg)	98.47 ± 10.79	93.70 ± 10.89	0.123
DBP (mmHg)	58.26 ± 9.80	59.08 ± 7.66	0.095
BMI (kg/m^2^)	16.4 ± 1.9	18.5 ± 2.8	0.002
Z-score BMI	-1.06 ± 0.9	0.68 ± 1.3	0.002
Hemoglobin (g/dL)	8.0 ± 0.9	13.1 ±1	<0.001
Hematocrit (%)	24 ± 3	39.8 ± 3.4	<0.001
MCV (fL)	90 ± 11	83 ± 5.2	0.004
MCHC (pg)	33.4 ± 4.5	33 ± 1.3	0.080
Leukocytes (×10^3^/L)	12.4 ± 3.8	7.1 ± 1.7	<0.001
Platelets (×10^3^/L)	455 ± 126	283 ± 46.5	<0.001
Reticulocytes (%)	7.4 ± 4.8	0.7 ± 0.2	<0.001
Lactate dehydrogenase (U/L)	1198 ± 567	368 ± 70	<0.001
SGOT (U/L)	49.8 ± 22	20.6 ± 4.2	<0.001
SGPT (U/L)	23.5 ± 12	14 ± 3.6	<0.001
Total bilirubin (mg/dL)	3.2 ± 1.9	0.59 ± 0.7	<0.001
Indirect bilirubin (mg/dL)	2.7 ± 1.9	0.46 ± 0.5	<0.001
hs-CRP (mg/L)^b^	2.02 (0.88–3.5)	0.42(0.17–1.63)	0.001
Total cholesterol (mg/dL)	122 ± 24	155 ± 26	<0.001
LDL-C (mg/dL)	69 ± 22	92 ± 23	<0.001
HDL-C (mg/dL)	33 ± 7	46 ± 12	<0.001
Triglycerides (mg/dL)	100 ± 39	74 ± 25	0.005
Glucose (mg/dL)	80 ± 10	87 ± 10	0.040

^a^Χ^2^,

^b^Median (P25 and P75) was obtained using Mann-Whitney test; Student’s t-test was used for other variables.

SBP, systolic blood pressure; DBP, diastolic blood pressure; SpO_2_, peripheral capillary oxygen saturation; MCV, mean cell volume; MCHC, mean cell hemoglobin concentration; SGPT, serum glutamic pyruvic transaminase; SGOT, serum glutamic oxaloacetic transaminase; hs-CRP, high sensitivity C-reactive protein; HDL-C, high-density lipoprotein cholesterol; LDL-C, low-density lipoprotein-cholesterol.

Moreover, BMI assessment demonstrated that up to 86% (n = 33) of SCA individuals and 64% (n = 16) of healthy individuals were eutrophic. The frequency of underweight subjects was higher in the SCA group compared to that in healthy controls (12.8% vs. 8%). Seven individuals (28%) from the control group and none in the SCA group were overweight. There was a predominance of non-whites in both groups (100% in the SCA and 96% in the control group).

We next examined the relationships between the use of HU and the sociodemographic factors and clinical and laboratory characteristics among SCA patients. We found no differences between SCA patients who did not use HU and SCA patients using HU, except for higher levels of lactate dehydrogenase (p = 0.03) and aspartate aminotransferase (p = 0.02) in the group of patients not using HU (Table 2). Variables related to SCA disease progression before the introduction of HU, such as total number of blood transfusions taken until the time of the study enrollment, were higher in the group of patients using HU (p = 0.001; Tables 2 and 3). Time from implementation of HU therapy was variable. The percentage of patients using HU for less than 6 months was 11%, in those who were taking HU for 6 months to 3 years was 83%, and for those who had more than 3 years undertaking HU was 16.7%.

**Table 2:** Laboratory characteristics of sickle cell anemia patients undergoing treatment with hydroxyurea (HU) at the study enrollment.

Parameter	No HU	HU	*P*-value
N	22	18	
Hb (g/dL)	7.8 ± 0.8	8.2 ± 0.95	0.17
HbF (g/dL)	6.3 ± 4.9	8.9 ± 6.9	0.23
MCV (fl)	86.7 ± 10	94.3 ± 11	0.03
Leukocytes (×10^3^/L)	12.7 ± 3.3	11.9 ± 4.4	0.50
Platelets (×10^3^/L)	451.6 ± 111	467.4 ± 146	0.70
Reticulocytes (%)	8.4 ± 5	6.1 ± 4	0.13
LDH (U/L)	1377 ± 603	991 ± 456	0.03
SGOT (U/L)	57 ± 23	41 ± 19	0.02
hs-CRP (mg/L)^a^^,^^b^	2.0(0.87–3.51)	2.2(0.87–4.11)	0.76
Total cholesterol (mg/dL)	122.6 ± 23	122 ± 26	0.88
LDL-C (mg/dL)	67 ± 24	69.6 ± 23	0.50
HDL-C (mg/dL)	34.6 ± 9.4	33 ± 5.7	0.70
Triglycerides (mg/dL)	104 ± 47	99.5 ± 28.6	0.40

^a^Median (p25 and p75);

^b^Mann-Whitney test

SGPT, serum glutamic pyruvic transaminase; SGOT, serum glutamic oxaloacetic transaminase; hs-CRP, high sensitivity C-reactive protein; HDL, high-density lipoprotein cholesterol; LDL-C, low-density lipoprotein-cholesterol; LDH, Lactate dehydrogenase; HbF, fetal hemoglobin; Student’s *t* test was used to compare variables expressed as mean ± SD

**Table 3:** Clinical and sociodemographic characteristics of sickle cell anemia patients undergoing treatment with hydroxyurea (HU) at the study enrollment.

Characteristics	No HU	HU	*P*-value
N	22	18	
Age (years)	12.9 ± 2.8	11.6 ± 3.1	0.17
Males, n (%)	13(63.6)	11(61)	0.87
BMI, (kg/m^2^)	16.8 ± 2	16 ± 1.3	0.18
BMI z-score	-1.0 ± 0.8	-1.1 ± 0.9	0.60
No. VOC attended to by physicians	0.7 ± 0.4	0.7 ± 0.5	0.97
No. VOC hospitalizations	0.8 ± 0.9	1.6 ± 1.7	0.20
No. ACS, previous year	0.36 ± 0.58	0.33 ± 0.76	0.88
ACS hospitalization period (days)	12.7 ± 22.2	17.4 ± 23.4	0.15
No. of blood transfusions, previous year	0.6 ± 1.13	2.4 ± 5	0.33
No. of blood transfusions before inclusion in the study	5.3 ± 6.0	14.1 ± 15.6	0.01
SpO_2_ (%)	94.5 ± 3.5	95.9 ± 2.0	0.14
No. of VOC with physical limitations	14 (63.6)	11 (61)	0.56
No. ACS	13 (59.1)	14 (77.8)	0.30
ACS oxygen use, n (%)	9 (40.9)	8 (44.4)	0.70
ACS blood transfusion, n (%)	9 (40.9)	9 (50)	0.70
Priapism, n (%)	3 (13.6)	1 (5.6)	0.60
Splenic sequestration, n (%)	2 (9.1)	4 (22)	0.38
Cholelithiasis, n (%)	7 (31.8)	4 (22.2)	0.37
Blood transfusions^a^, n (%)	18 (81.8)	16 (88.9)	0.67

Data represent mean values with standard deviations or frequencies. Fisher's exact test and Student’s *t* test were performed. VOC, vaso-occlusive crisis; ACS, acute chest syndrome; SpO_2_%, peripheral capillary oxygen saturation.

^a^Percentage of patients who received blood transfusion before inclusion in the study.

**Fig 1:**
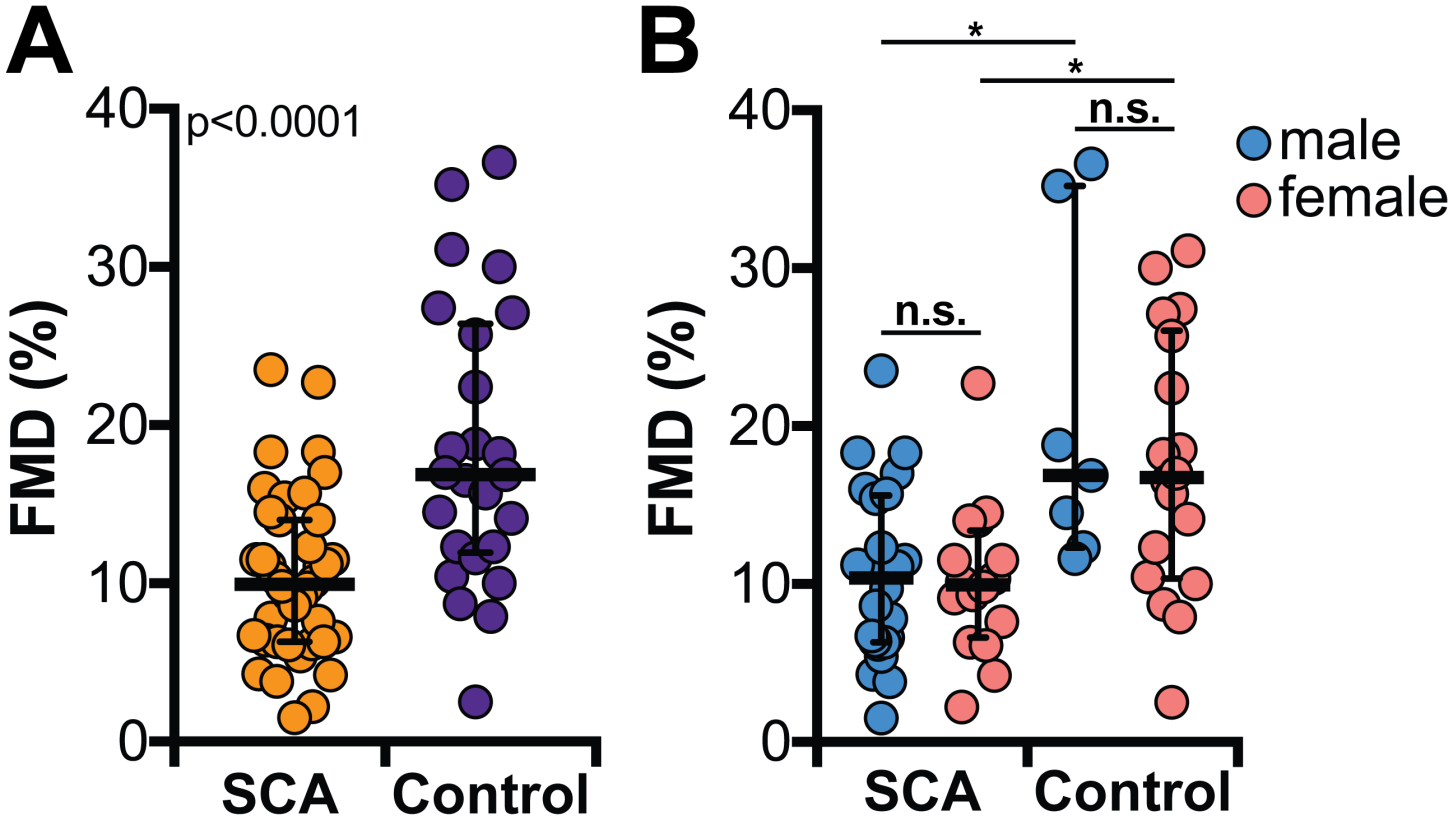
Flow-mediated vasodilation (FMD) values are reduced in sickle cell anemia patients compared to that in healthy controls independent of gender. Lines represent median and interquartile values. Data were compared using the Mann-Whitney *U* test (A) or Kruskal-Wallis test with Dunn’s multiple comparisons ad hoc test. *p<0.05; n.s. = non-significant.

Moreover, we examined endothelial function using FMD in SCA patients and compared to that from healthy controls. We observed that SCA patients exhibited on average lower FMD values than that in the healthy individuals (Fig 1A). Our primary analyses revealed that there was a predominance of males in the SCA group (Table 1). At first glance, this finding could be potentially influencing the lower average of FMD in the SCA group. To directly test this hypothesis, we compared FMD values between male and females in both study groups. We found that there were no differences in FMD values between male and female study participants within a given study group, and that FMD values were found to be reduced in SCA patients irrespective of gender (Fig 1B).

**Fig 2:**
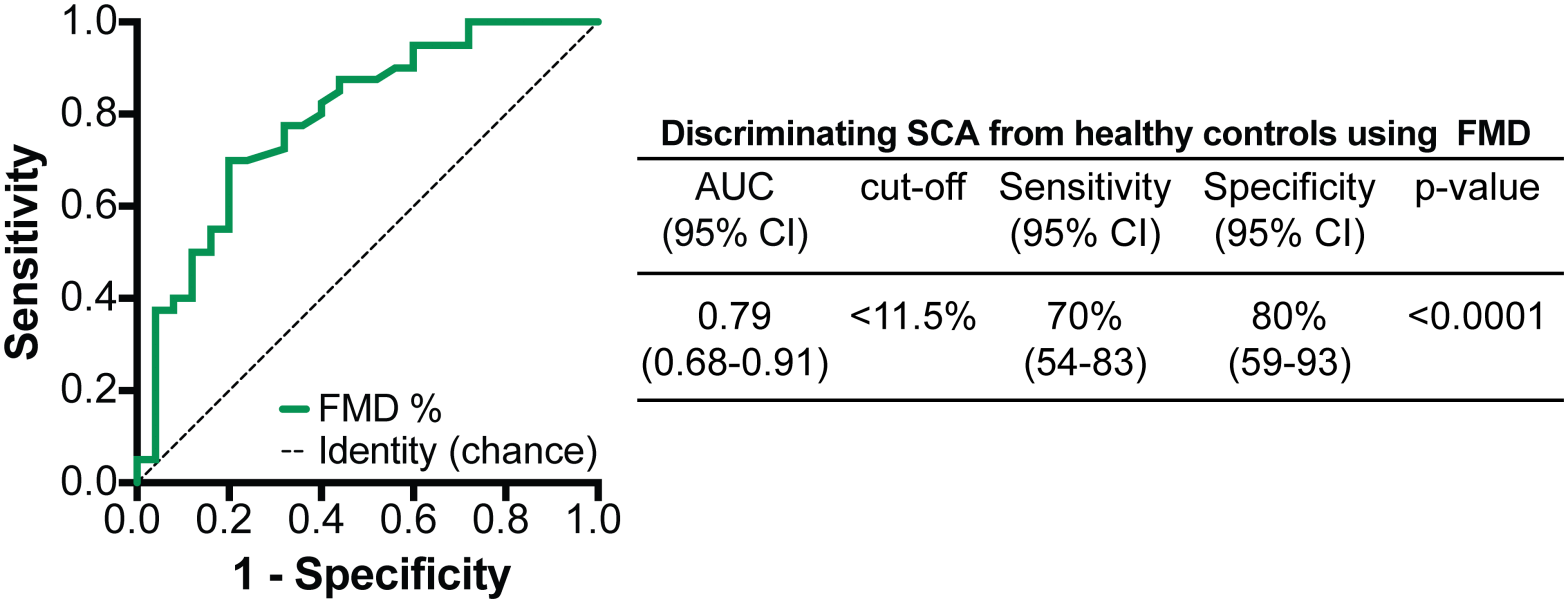
Receiver Operator Characteristics (ROC) curve analysis of FMD values to differentiate SCA from healthy controls. AUC, area under the curve; CI, confidence interval.

No differences were observed in the FMD between patients presenting with BMI Z-scores ≤+1 or >+1 (13% ± 7.5% vs. 17% ± 9.5%, p = 0.16). We next employed Receiver Operator Characteristics (ROC) curve analysis to test whether FMD as measure of endothelial function could be used to discriminate SCA from healthy controls. We found that a FMD value below 11.5% exhibited a sensitivity of 70% and specificity of 80% for distinguishing SCA from controls (Fig 2). These findings suggest that low FMD values are strongly associated with SCA, reinforcing the idea that endothelial dysfunction is a hallmark of this disease.

**Fig 3:**
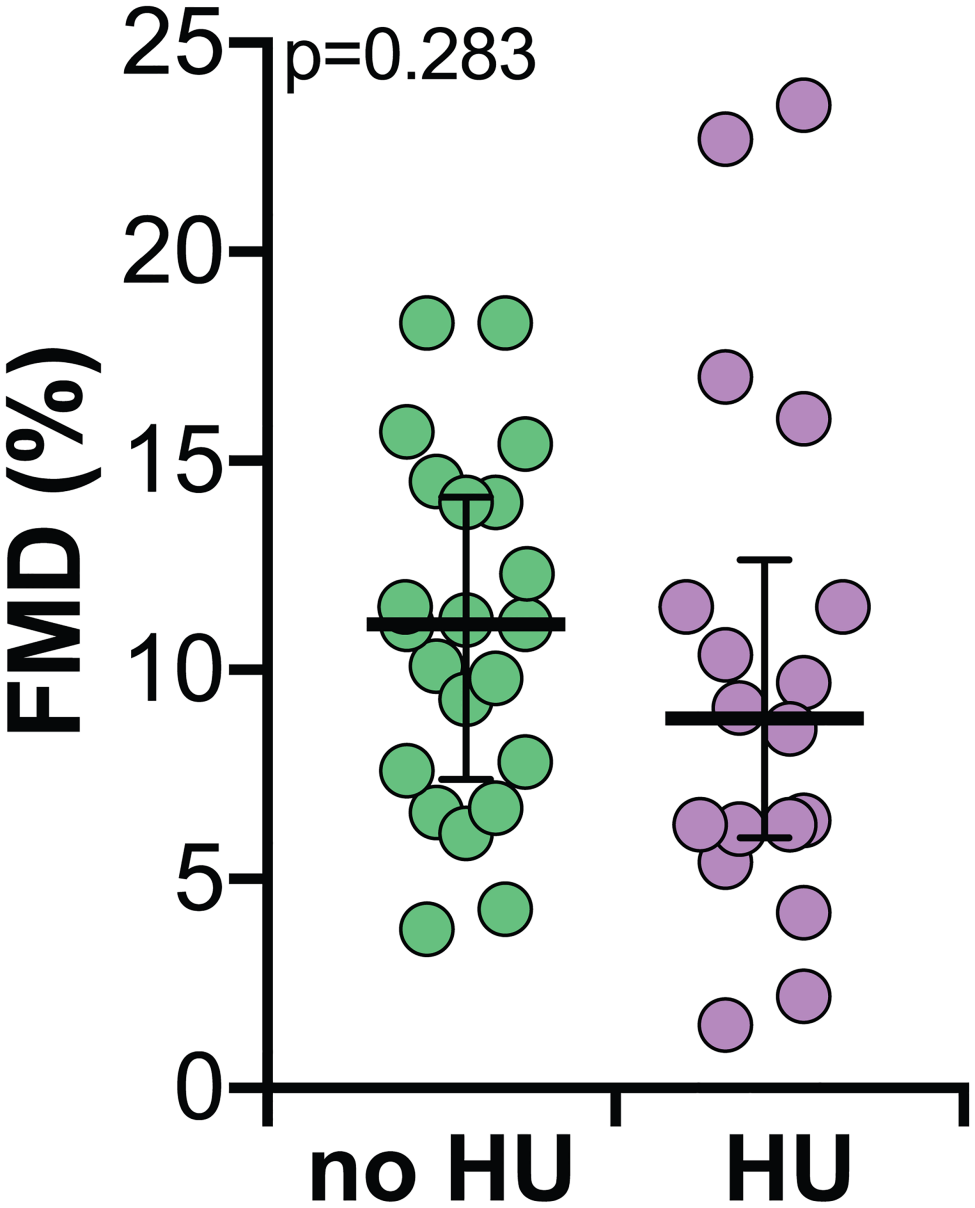
Comparison of flow-mediated vasodilation (FMD) values in SCA patients undergoing or not hydroxyurea therapy. Lines represent median and interquartile values. Data were compared using the Mann-Whitney *U* test.

Furthermore, we compared FMD values in SCA persons who were taking HU to that from those who were not taking this treatment and found that such values were undistinguishable between these patient groups (Fig 3). The small size of such comparison groups precludes a definite conclusion about the HU use and its effects on FMD values.

In a preliminary assessment, we examined correlations between FMD and clinical and laboratory variables in the HbSS individuals and found a negative association with number of ACS episodes observed over the last year (r = - 0.343, p = 0.030).

**Fig 4:**
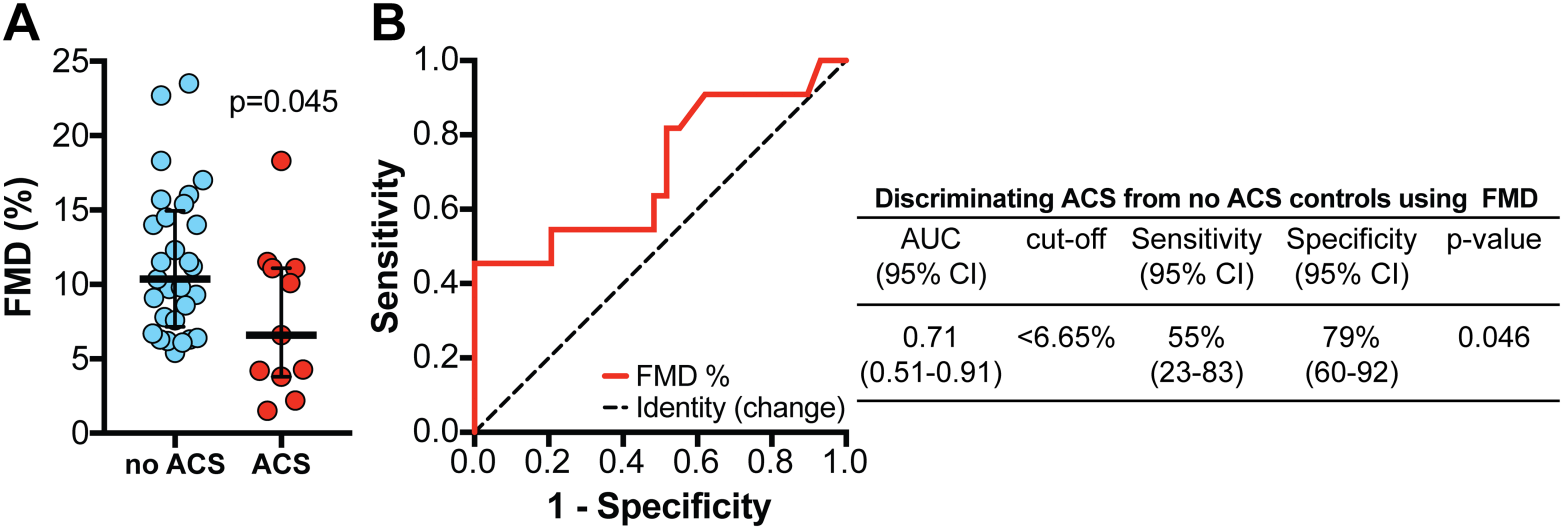
Using flow-mediated vasodilation (FMD) values in SCA patients to identify individuals with ACS history. (A) FMD values were compared between the groups of ACS and controls using the Mann-Whitney *U* test. Lines represent median values and interquartile ranges. Receiver Operator Characteristics (ROC) curve analysis was employed to test the accuracy of FMD values to discriminate between individuals with ACS history and controls.

Based on previous publications, the 10^th^ percentile of FMD in the healthy group (8.39%) could be considered as cutoff value used to stratify patients based on endothelial dysfunction [27,28]. Using this criterion, we found no significant differences in the means of clinical and laboratory variables between the groups of SCA patients categorized as presenting with or without endothelial dysfunction. Furthermore, we tested whether FMD values could be used to identify SCA patients with endothelial dysfunction by means of ACS episodes. We found that SCA patients with history of ACS in the last year exhibited significantly lower FMD values compared to those who had no ACS (Fig 4A). In addition, ROC curve analysis revealed that FMD values can be used to distinguish ACS from no ACS history in SCA patients with an accuracy of 71% and specificity of up to 79% (Fig 4B). SCA patients presenting with FMD values below 6.65% exhibited higher odds for having ACS history, after adjustment for age and gender (OR: 1.45, 95% confidence interval: 1.02–3.45, p = 0.023). These observations suggest that low FMD values are strongly associated with history of ACS in the last year in SCA patients. Interestingly, at the study entry, out of 37 individuals who had VOC, 26 (70.3%) also reported history of ACS. This finding suggests that these two clinical outcomes may be related in the context of SCA.

## Discussion

Sickle cell anemia is as a vascular disease that involves genetic, environmental and biological factors. This disease has been described to activate endothelial cells, ultimately compromising organs and systems [29,30]. Some manifestations related to vascular activation/damage such as pulmonary hypertension, ACS and stroke have been associated with higher mortality, more hospitalizations, and impaired quality of life in SCA [30–32]. It is possible that changes in endothelial function are reflecting the degree of vascular activation/damage and its related complications. Few studies have demonstrated endothelial dysfunction in children and adolescents with SCA by evaluating FMD, and the reported results are discordant [33,34]. FMD as measure of endothelial function has been widely used, is noninvasive and has low cost, justifying its use in the present study. Furthermore, to the best of our knowledge, no previous study has examined FMD values among SCA patients undergoing or not therapy with HU.

In this study, FMD values were significantly lower in children and adolescents with stable SCA than that in healthy subjects, suggesting a diminished vasodilation capacity in such patients due to the impairment of the endothelial function in SCA. This finding is relevant and leads us to hypothesize that vascular activation/damage in SCA may occur during early stages, although this specific question has not been directly tested in our study. It has already been shown that, in SCA patients, thrombus formation induced by clusters of polymerized erythrocytes, leukocytes, and platelets, together with release of free hemoglobin via intravascular hemolysis, are all associated with endothelial activation. These events ultimately lead to increase in NO consumption while decreasing its production, resulting in deterioration of the endothelial capacity to maintain normal vascular tone [5,10]. Whether the low values of FMD detected in our SCA directly reflect the degree of reduction of NO bioavailability in the blood is unknown and future studies are warranted to answer this question.

Our study groups were matched by age, but not gender. We found increased frequency of female individuals in the group of healthy controls compared to that in the SCA group. Previous reports have indicated that there is an age-decline in FMD values and that healthy male individuals display lower FMD values than age-matched females [35,36]. Our results clearly indicate that FMD values are lower in SCA patients compared to healthy, age-matched, controls independent of gender. Larger studies are necessary to validate this finding.

In SCA, the implementation of HU therapy is usually reserved for patients exhibiting more severe disease [37]. An exploratory analysis performed in the present study revealed that SCA patients who were taking HU exhibited only slight and non-statistically significant decreases in FMD values compared to those patients not undergoing this therapy. It is possible that the small number of patients and substantial dispersion in FMD values prevented us to detect significant differences. This preclude us to trace a definite conclusion between this therapy and FMD values. Moreover, additional studies are necessary to better examine the effects of HU therapy on FMD values, as this drug has been described to increase circulating concentrations of fetal hemoglobin (HbF) and to decreased expression of endothelial adhesion molecules, aside from increasing NO production, all of which are linked to preservation of vascular integrity in SCA [34,38]. The idea that HU may improve FMD is worth of study, but will need additional prospective investigations better delineated to specifically test this hypothesis.

Previous studies have established a cutoff value for FMD (defined as the 10^th^ percentile value) to infer endothelial dysfunction in healthy children [27,28,39]. Using this approach in our study population, we found the value of 8.4%. After stratification of our SCA patient group according to the occurrence of endothelial dysfunction, we found no significant differences in distribution of clinical and laboratory variables. However, when we stratified the SCA group using a FMD cutoff established in the ROC curve analysis, we observed a substantial increase in odds of ACS in patients with lower FMD values, suggesting that such patients exhibit significant endothelial dysfunction. This observation led us to speculate that FMD values below 6.65% may be associated with more severe clinical manifestations of SCA, suggesting that the FMD values may be inversely associated with the degree of vascular activation/damage. Additional mechanistic studies are warranted to specifically test this hypothesis.

In our analyses, FMD values exhibited a negative correlation with the number of ACS episodes reported. Two or more episodes of ACS in one year are considered a criteria for initiating HU therapy because of its association with SCA clinical severity [40,41]. In the ACS, hemolysis and endothelial damage are observed in addition to vaso-occlusion, thrombus formation and inflammation [41]. Moreover, this syndrome is accompanied by increased inflammation-driven ROS production and decreased bioavailability of NO [41]. In this setting, reduction in NO bioavailability [42] contributes to vasoconstriction and favors the adhesion of sickle erythrocytes to the endothelium, potentially exacerbating vaso-occlusive process, and hypoxia in the pulmonary microvasculature. In the multivariate analysis, the number of ACS episodes points to an independent factor associated with increased vascular damage. Although cross-sectional studies cannot establish a cause-effect relationship, our findings together with published mechanisms involved in the pathophysiology of ACS allow us to hypothesize that endothelial dysfunction may favor development of SCA complications. Of note, ACS is the second leading cause of hospitalization in SCA children over 2 years of age, the leading cause of admission to intensive care units and the top cause of mortality in individuals with SCA [31,32].

This study has several limitations. The endothelial function was assessed using an image-based methodology and is depended on the ability of the person operating the procedure. Nevertheless, several studies have demonstrated that FMD measurement produces reliable and accurate results, aside from being a safe and low-cost procedure [39]. There is no consensus about which FMD value should be used to infer increased or reduced vascular damage in children and adolescents with SCA, which makes necessary to compare with healthy control groups. We have not measured free hemoglobin, free heme, free iron and other more sensitive markers of hemolysis-associated inflammation and potential vascular damage than FMD values. Our group is currently performing mechanistic studies in larger patient populations to compare these markers.

## Conclusion

Children and adolescents with SCA have impaired endothelial function. The study was underpowered to detect differences between patients undergoing or not HU therapy, and a potential association between HU use and FMD values deserves further investigation.

## References

[1] Ware RE (2013) Is sickle cell anemia a neglected tropical disease? PLoS Negl Trop Dis 7: e2120 doi: 10.1371/journal.pntd.0002120 23750287 10.1371/journal.pntd.0002120 PMC3671937

[2] Steinberg MH (2006) Pathophysiologically based drug treatment of sickle cell disease. Trends Pharmacol Sci 27: 204–210. doi: 10.1016/j.tips.2006.02.007 16530854 10.1016/j.tips.2006.02.007

[3] Stuart MJ, Nagel RL (2004) Sickle-cell disease. Lancet 364: 1343–1360. 15474138 10.1016/S0140-6736(04)17192-4

[4] Steinberg MH (2005) Predicting clinical severity in sickle cell anaemia. Br J Haematol 129: 465–481. 15877729 10.1111/j.1365-2141.2005.05411.x

[5] Kato GJ, Gladwin MT, Steinberg MH (2007) Deconstructing sickle cell disease: reappraisal of the role of hemolysis in the development of clinical subphenotypes. Blood Rev 21: 37–47. doi: 10.1016/j.blre.2006.07.001 17084951 10.1016/j.blre.2006.07.001 PMC2048670

[6] Rother RP, Bell L, Hillmen P, Gladwin MT (2005) The clinical sequelae of intravascular hemolysis and extracellular plasma hemoglobin: a novel mechanism of human disease. JAMA 293: 1653–1662. doi: 10.1001/jama.293.13.1653 15811985 10.1001/jama.293.13.1653

[7] Furchgott RF, Zawadzki JV (1980) The obligatory role of endothelial cells in the relaxation of arterial smooth muscle by acetylcholine. Nature 288: 373–376. 6253831 10.1038/288373a0

[8] Verma S, Buchanan MR, Anderson TJ (2003) Endothelial function testing as a biomarker of vascular disease. Circulation 108: 2054–2059. doi: 10.1161/01.CIR.0000089191.72957.ED 14581384 10.1161/01.CIR.0000089191.72957.ED

[9] Reiter CD, Wang X, Tanus-Santos JE, Hogg N, Cannon RO 3rd, Schechter AN, et al (2002) Cell-free hemoglobin limits nitric oxide bioavailability in sickle-cell disease. Nat Med 8: 1383–1389. 12426562 10.1038/nm1202-799

[10] Gladwin MT (2006) Deconstructing endothelial dysfunction: soluble guanylyl cyclase oxidation and the NO resistance syndrome. J Clin Invest 116: 2330–2332. doi: 10.1172/JCI29807 16955136 10.1172/JCI29807 PMC1555666

[11] Gladwin MT, Sachdev V, Jison ML, Shizukuda Y, Plehn JF, Minter K, et al (2004) Pulmonary hypertension as a risk factor for death in patients with sickle cell disease. N Engl J Med 350: 886–895. 14985486 10.1056/NEJMoa035477

[12] Wood KC, Hsu LL, Gladwin MT (2008) Sickle cell disease vasculopathy: a state of nitric oxide resistance. Free Radic Biol Med 44: 1506–1528. doi: 10.1016/j.freeradbiomed.2008.01.008 18261470 10.1016/j.freeradbiomed.2008.01.008

[13] Moens AL, Goovaerts I, Claeys MJ, Vrints CJ (2005) Flow-mediated vasodilation: a diagnostic instrument, or an experimental tool? Chest 127: 2254–2263. doi: 10.1378/chest.127.6.2254 15947345 10.1378/chest.127.6.2254

[14] Vita JA, Keaney JF Jr. (2002) Endothelial function: a barometer for cardiovascular risk? Circulation 106: 640–642. 12163419 10.1161/01.cir.0000028581.07992.56

[15] Pearson TA, Mensah GA, Alexander RW, Anderson JL, Cannon RO 3rd, Criqui M, et al (2003) Markers of inflammation and cardiovascular disease: application to clinical and public health practice: A statement for healthcare professionals from the Centers for Disease Control and Prevention and the American Heart Association. Circulation 107: 499–511. 12551878 10.1161/01.cir.0000052939.59093.45

[16] Organization WH (2014) Child growth standards: length/height-for-age, weight-for-age, weight-for-length, weight-for-height and body mass index-for-age.

[17] de Montalembert M, Aggoun Y, Niakate A, Szezepanski I, Bonnet D (2007) Endothelial-dependent vasodilation is impaired in children with sickle cell disease. Haematologica 92: 1709–1710. doi: 10.3324/haematol.11253 18055999 10.3324/haematol.11253

[18] Wang WC, Ware RE, Miller ST, Iyer RV, Casella JF, Minniti CP, et al (2011) Hydroxycarbamide in very young children with sickle-cell anaemia: a multicentre, randomised, controlled trial (BABY HUG). Lancet 377: 1663–1672. doi: 10.1016/S0140-6736(11)60355-3 21571150 10.1016/S0140-6736(11)60355-3 PMC3133619

[19] Charache S, Scott JC, Charache P (1979) "Acute chest syndrome" in adults with sickle cell anemia. Microbiology, treatment, and prevention. Arch Intern Med 139: 67–69. 32855

[20] Vichinsky EP, Styles LA, Colangelo LH, Wright EC, Castro O, Nickerson B (1997) Acute chest syndrome in sickle cell disease: clinical presentation and course. Cooperative Study of Sickle Cell Disease. Blood 89: 1787–1792. 9057664

[21] Ballas SK, Kesen MR, Goldberg MF, Lutty GA, Dampier C, Osunkwo I, et al (2012) Beyond the definitions of the phenotypic complications of sickle cell disease: an update on management. ScientificWorldJournal 2012: 949535 doi: 10.1100/2012/949535 22924029 10.1100/2012/949535 PMC3415156

[22] Gill FM, Sleeper LA, Weiner SJ, Brown AK, Bellevue R, Grover R, et al (1995) Clinical events in the first decade in a cohort of infants with sickle cell disease. Cooperative Study of Sickle Cell Disease. Blood 86: 776–783. 7606007

[23] Corretti MC, Anderson TJ, Benjamin EJ, Celermajer D, Charbonneau F, Creager MA, et al (2002) Guidelines for the ultrasound assessment of endothelial-dependent flow-mediated vasodilation of the brachial artery: a report of the International Brachial Artery Reactivity Task Force. J Am Coll Cardiol 39: 257–265. 11788217 10.1016/s0735-1097(01)01746-6

[24] Ladeia AM, Ladeia-Frota C, Pinho L, Stefanelli E, Adan L (2005) Endothelial dysfunction is correlated with microalbuminuria in children with short-duration type 1 diabetes. Diabetes Care 28: 2048–2050. 16043758 10.2337/diacare.28.8.2048

[25] Sampaio RR, Ladeia AM, Meneses RB, Lima Mde L, Guimaraes AC (2013) C-reactive protein is not correlated with endothelial dysfunction in overweight and obese women. J Clin Med Res 5: 294–299. doi: 10.4021/jocmr1418w 23864919 10.4021/jocmr1418w PMC3712885

[26] Ladeia AM, Sampaio RR, Hita MC, Adan LF (2014) Prognostic value of endothelial dysfunction in type 1 diabetes mellitus. World J Diabetes 5: 601–605. doi: 10.4239/wjd.v5.i5.601 25317238 10.4239/wjd.v5.i5.601 PMC4138584

[27] Jarvisalo MJ, Ronnemaa T, Volanen I, Kaitosaari T, Kallio K, Hartiala JJ, et al (2002) Brachial artery dilatation responses in healthy children and adolescents. Am J Physiol Heart Circ Physiol 282: H87–92. 11748051 10.1152/ajpheart.2002.282.1.H87

[28] Andrade ZM, Carvalhaes JT, Taddei JA, Christofalo DM, Ajzen SA (2005) [Endothelial function of normotensive adolescents with no risk factors for arterial hypertension]. J Pediatr (Rio J) 81: 395–399. 16247542 10.2223/1377

[29] Steinberg MH, Adewoye AH (2006) Modifier genes and sickle cell anemia. Curr Opin Hematol 13: 131–136. doi: 10.1097/01.moh.0000219656.50291.73 16567954 10.1097/01.moh.0000219656.50291.73

[30] Steinberg MH, Sebastiani P (2012) Genetic modifiers of sickle cell disease. Am J Hematol 87: 795–803. doi: 10.1002/ajh.23232 22641398 10.1002/ajh.23232 PMC4562292

[31] Platt OS, Thorington BD, Brambilla DJ, Milner PF, Rosse WF, Vichinsky E, et al (1991) Pain in sickle cell disease. Rates and risk factors. N Engl J Med 325: 11–16. doi: 10.1056/NEJM199107043250103 1710777 10.1056/NEJM199107043250103

[32] Thomas AN, Pattison C, Serjeant GR (1982) Causes of death in sickle-cell disease in Jamaica. Br Med J (Clin Res Ed) 285: 633–635. 10.1136/bmj.285.6342.633 PMC1499426 6819042

[33] Hadeed K, Hascoet S, Castex MP, Munzer C, Acar P, Dulac Y (2015) Endothelial Function and Vascular Properties in Children with Sickle Cell Disease. Echocardiography 32: 1285–1290. doi: 10.1111/echo.12851 25470331 10.1111/echo.12851

[34] Cartron JP, Elion J (2008) Erythroid adhesion molecules in sickle cell disease: effect of hydroxyurea. Transfus Clin Biol 15: 39–50. doi: 10.1016/j.tracli.2008.05.001 18515167 10.1016/j.tracli.2008.05.001

[35] Herrington DM, Fan L, Drum M, Riley WA, Pusser BE, Crouse JR, et al (2001) Brachial flow-mediated vasodilator responses in population-based research: methods, reproducibility and effects of age, gender and baseline diameter. J Cardiovasc Risk 8: 319–328. 11702039 10.1177/174182670100800512

[36] Celermajer DS, Sorensen KE, Spiegelhalter DJ, Georgakopoulos D, Robinson J, Deanfield JE (1994) Aging is associated with endothelial dysfunction in healthy men years before the age-related decline in women. J Am Coll Cardiol 24: 471–476. 8034885 10.1016/0735-1097(94)90305-0

[37] Ministério da Saúde SdAàS (2002) Protocolo clínico e diretrizes terapêuticas—Doença Falciforme.

[38] Lou TF, Singh M, Mackie A, Li W, Pace BS (2009) Hydroxyurea generates nitric oxide in human erythroid cells: mechanisms for gamma-globin gene activation. Exp Biol Med (Maywood) 234: 1374–1382. 19657070 10.3181/0811-RM-339 PMC3827858

[39] Jarvisalo MJ, Raitakari OT (2005) Ultrasound assessment of endothelial function in children. Vasc Health Risk Manag 1: 227–233. 17319108 PMC1993949

[40] Cançado RD, Clarisse L, ANgulo IL, Araújo PIC, Jesus JA (2009) Protocolo clínico e diretrizes terapêuticas para uso de hidroxiureira da doença falciforme. Revista Barsileira de Hematologia e Hemoterapia 31: 361–366.

[41] Gualandro SFM, Fonseca GHH, Gualandro DM (2007) Cardiopulmonary complications of sickle cell disease. Revista Barsileira de Hematologia e Hemoterapia 29: 291–298.

[42] El-Ghamrawy MK, Hanna WM, Abdel-Salam A, El-Sonbaty MM, Youness ER, Adel A (2014) Oxidant-antioxidant status in Egyptian children with sickle cell anemia: a single center based study. J Pediatr (Rio J) 90: 286–292. 24508012 10.1016/j.jped.2013.09.005

